# Differential susceptibilities of *Anopheles albimanus* and *Anopheles stephensi* mosquitoes to ivermectin

**DOI:** 10.1186/s12936-018-2296-3

**Published:** 2018-04-03

**Authors:** Staci M. Dreyer, Kelsey J. Morin, Jefferson A. Vaughan

**Affiliations:** Department of Biology, University of North Dakota, 10 Cornell Street, Stop 9019, Grand Forks, ND 58202-9019 USA

**Keywords:** *Anopheles albimanus*, *Anopheles stephensi*, Endectocide, Ivermectin, Vector control

## Abstract

**Background:**

Vector control is a crucial element of anti-malaria campaigns and works best when there is a thorough knowledge of the biology and behaviour of the *Anopheles* vector species responsible for transmitting malaria within a given locale. With the push to eradicate malaria stronger than ever, there is a growing need to develop and deploy control strategies that exploit the behavioural attributes of local vector species. This is especially true in regions where the vectors are exophagic (i.e., prefer to bite outdoors), exophilic (i.e., prefer to remain outdoors), and zoophagic (i.e., as likely to feed on non-humans as humans). One promising strategy targeting vectors with these behavioural traits is the administration of avermectin-based endectocides, such as ivermectin, to humans and livestock. When ingested in a blood meal, ivermectin has been shown to reduce mosquito survivorship and fecundity in a number of *Anopheles* species. In this study, the relative toxicity of ivermectin was compared between two zoophagic, exophilic malaria vectors—*Anopheles albimanus* and *Anopheles stephensi*.

**Results:**

Toxicity of ivermectin was assessed using membrane feedings, intrathoracic injections, and mosquito feedings on treated mice. When ingested in a blood meal, ivermectin was much less toxic to *An. albimanus* (4-day oral LC_50_ = 1468 ng/ml) than to *An. stephensi* (4-day oral LC_50_ = 7 ng/ml). However when injected into the haemocoel of *An. albimanus*, ivermectin was much more toxic (3-day parenteral LC_50_ = 188 ng/ml). Because the molecular targets of ivermectin (i.e., glutamate-gated chloride channels) reside outside the midgut in nerves and muscles, this suggests that ingested ivermectin was not readily absorbed across the midgut of *An. albimanus*. In contrast, ivermectin was considerably more toxic to *An. stephensi* when ingested (4-day oral LC_50_ = 7 ng/ml) than when injected (3-day parenteral LC_50_ = 49 ng/ml). This suggests that metabolic by-products from the digestion of ivermectin may play a role in the oral toxicity of ivermectin to *An. stephensi*. Blood meal digestion and subsequent oviposition rates were significantly hindered in both species by ingested ivermectin but only at concentrations at or above their respective oral LC_50_ concentrations. To test mosquitocidal activity of ivermectin in a live host system, two groups of three mice each received subcutaneous injections of either ivermectin (600 µg/kg BW) or saline (control). One day after injection, the ivermectin-treated mice (n = 3) exhibited significant mosquitocidal activity against both *An. stephensi* (85% mortality vs 0% in control-fed) and, to a lesser degree, *An. albimanus* (44% mortality vs 11% in control-fed). At 3 days, the mosquitocidal activity of ivermectin-treated mice waned and was effective only against *An. stephensi* (31% mortality vs 3% in control-fed).

**Conclusions:**

Ivermectin was not uniformly toxic to both *Anopheles* species. Previous studies indicate that ivermectin is a good choice of endectocide to use against malaria vectors in southeast Asia and Africa. However, these data suggest that ivermectin may not be the optimal endectocide to use in Central America or the Caribbean where *An. albimanus* is a major malaria vector species. If endectocides are to be used to help eradicate malaria, then additional efficacy data will be needed to define the activity of specific endectocides against the major malaria vector species of the world.

## Background

Malaria remains a major public health problem throughout the tropics [1] and is transmitted by *Anopheles* mosquitoes. Vector control is an essential element of malaria control programmes. Throughout sub-Saharan Africa, indoor residual spraying (IRS) and insecticide treated bed-nets (ITN) have been successful vector control tactics because the primary vector species, *Anopheles gambiae* feed almost exclusively on people sleeping in their houses at night and rest inside the house after blood-feeding [2–4]. Other important malaria vector species in Africa (e.g., *Anopheles arabiensis*), Asia (e.g., *Anopheles stephensi*) and the neotropics (e.g., *Anopheles albimanus*) are exophagic (i.e., prefer to bite outdoors), exophilic (i.e., prefer to rest outdoors), and/or zoophagic (i.e., as likely to feed on non-humans as humans) [5]. Effective control of *Anopheles* species with these behavioural characteristics requires alternative tactics. The primary non-human blood sources for zoophagic malaria vectors are peridomestic livestock (cattle, goats). Livestock represent mosquito blood sources that humans can manage and control. It is logical that any comprehensive strategy against zoophagic malaria vectors should include some sort of livestock management component. One tactic to control zoophagic malaria vectors is to treat livestock with ivermectin.

Ivermectin is a lipophilic drug that belongs to the avermectin class of macrocyclic lactone compounds. Ivermectin acts as an endectocide (i.e., kills both endoparasites and ectoparasites). Ivermectin binds to and activates the glutamate-gated chloride channels of nerve and muscle cells of a wide variety of nematode and arthropod species, causing uncontrolled influx of chloride ions into the cells and leading to paralysis and death of the organism [6]. Ivermectin has a high safety profile in humans and livestock [7] and is one of the most commonly-used anti-helminthic drug in the livestock industry to control intestinal nematodes.

The large-scale administration of oral ivermectin to people has become a cornerstone in the global eradication campaign against human onchocerciasis and lymphatic filariasis [8, 9]. The recommended dose of ivermectin used to treat humans for filarial infection [i.e., 150 mg/kg body weight (BW)] yields peak plasma concentrations of ca. 40–45 ng/ml [10]. At these plasma concentrations, ivermectin can significantly reduce the survival of *Anopheles* mosquitoes that ingest treated blood. *Anopheles* species shown to be susceptible to ivermectin at these concentrations include major vectors from Africa (*An. gambiae* [11–13], *An. arabiensis* [14]), Southeast Asia (*Anopheles campestris*, *Anopheles dirus*, *Anopheles minimus*, *Anopheles swandwongporni* [15]) and Latin America (*Anopheles aquasalis* [16], *Anopheles darlingi* [17]). Pilot field trials in western Africa indicate that the mass drug administration of ivermectin against onchocerciasis and lymphatic filariasis can simultaneously reduce survival of *An. gambiae* and local malaria transmission [18, 19].

Similarly, ivermectin treatment of livestock could theoretically reduce vector abundance and lower malaria transmission [20, 21]. At the same time, treatment of herds may also reduce tick and intestinal worm burdens, leading to increases in livestock weight gain, milk production, and ultimately increases in the health and economic prosperity of livestock owners. However, it is currently unknown if all species of *Anopheles* vectors are equally susceptible to ivermectin. This study compared the relative toxicity of ivermectin to two known malaria vectors: *Anopheles (Nyssorhynchus) albimanus*, a major vector along the coastal regions of Mexico, Central America, the Caribbean, and northern South America [22], and *Anopheles (Cellia) stephensi*, an important malaria vector in southern and western Asia [23]. Both species are highly zoophagic, exophilic and exophagic, making them appropriate species to target with a strategy involving ivermectin treatment of livestock.

## Methods

### Mosquitoes

Laboratory colonies of *An. albimanus* STECL strain and *An. stephensi* STE2 strain were obtained as eggs through BEI Resources (Manassas, VA USA). Mosquitoes were reared in the University of North Dakota insectary at a photoperiod of 12-h light:12-h dark and a temperature of 26 °C. Eggs were hatched in trays of dechlorinated water. Larvae were fed fish food (Tetra Pond Sticks, Tetra, Blacksburg, VA USA) ground to a fine powder in an electric coffee grinder. Pupae were placed into ca. 28 l wire mesh cages to emerge. Adults were given access to water and a sugar source. Female mosquitoes 3–7 days old for use in toxicity studies were aspirated from rearing cages into smaller (ca. 0.5 l), cylindrical cardboard containers with mesh tops at a density of 15–40 mosquitoes per cage.

### Membrane feeding

For each membrane feeding trial, age-matched cohorts of *An. albimanus* and *An. stephensi* were blood fed simultaneously on identical ivermectin preparations. For each feeding trial, stock solutions were prepared fresh by diluting powdered technical grade ivermectin (Sigma-Aldrich, St. Louis MO, USA) into dimethyl sulfoxide (DMSO) at a concentration of 2 mg/ml. The stock solution was diluted to starting concentrations in phosphate-buffered saline (PBS). From that, serial dilutions were made in 1.5 ml polypropylene microfuge tubes containing whole bovine blood with sodium heparin (10 U/ml, Pel-Freez Biologicals, Rodgers AR, USA) for a total volume of 1 ml. A control group (i.e., 10 µl PBS added to 990 µl blood) was included with each trial. Prior to feeding, the tubes were inverted a minimum of three times to mix the solutions and kept in warm water. Natural sausage casing (Dewied International, San Antonio TX, USA) was rinsed thoroughly to remove salt preservative and cut to fit across the bottom of glass membrane feeders. Feeders were connected to one another with rubber tubing and heated water (ca. 37 °C) was circulated to warm the feeders. Membrane feeders were placed one per cage, and the blood-ivermectin mixtures were pipetted into the feeders. Mosquitoes were allowed to feed for ca. 30 min. Unfed mosquitoes were removed. Engorged mosquitoes were maintained in the insectary and provided with cotton soaked in a 10% sucrose solution. Cages were checked every day. Dead mosquitoes were counted and removed. For most trials, the number of surviving mosquitoes was counted at 4 days. In one trial, survival counts were extended to 5 days to determine if mortality had stabilized. By day 5, the rate of mortality stabilized and became asymptotic. Thus, 4 days was selected as an appropriate time on which to base LC_50_ determinations for oral toxicity. After preliminary trials to determine an appropriate range of concentrations, feedings were replicated five times using 4–5 ivermectin concentrations, plus a control, for each feeding trial.

### Intrathoracic injection

Mosquitoes were immobilized by chilling (− 20 °C for ca. 20 s), then quickly transferred to filter paper onto a chill table (BioQuip, Rancho Dominguez, CA, USA). To perform injections, a semi-automated micro-injector (TriTech Research, Los Angeles CA, USA) was fitted with fine-tipped glass needles made with a micropipette puller (World Precision, Sarasota FL, USA). A stock solution was prepared as described above and from that, serial dilutions were made in 1.5 ml polypropylene microfuge tubes containing an insect salt solution [24]. Mosquitoes were injected with 0.4 µl of solution into the thorax, near the base of the wing. Mosquitoes were then placed into appropriately labeled recovery containers. Injected mosquitoes were provided 10% sucrose solution and checked daily for 3 days. Dead mosquitoes were counted and removed over the course of 3 days, at which time the surviving mosquitoes were counted. In one trial, survival counts were extended to 4 days to determine if mortality had stabilized. By day 4, the rate of mortality stabilized and became asymptotic. Thus, 3 days was selected as an appropriate time on which to base LC_50_ determinations for parenteral toxicity. After preliminary trials to determine an appropriate range of concentrations, injections were replicated three times for *An. stephensi* and four times for *An*. *albimanus*, using 4–5 ivermectin concentrations plus a control at each injection trial.

### Fecundity

Mosquitoes were fed with membrane feeders on blood containing various doses of ivermectin, as described previously. *Anopheles stephensi* were given a single experimental blood meal and monitored for egg production. However during colony rearing of *An. albimanus,* it was noted that this species required more than one blood feeding in order to produce sufficient eggs to maintain the colony. To accurately assess the effect of ivermectin on *An. albimanus* fecundity, it was necessary that control-fed females undergo full gonotrophic development. Therefore, *An. albimanus* were first given a normal blood meal (i.e., cow blood with no drugs). Unfed mosquitoes were removed and engorged *An. albimanus* were held for 2 days without oviposition sites. The mosquitoes were then offered a second, experimental blood meal. Unfed mosquitoes were removed. One day after experimental blood meals, fully engorged mosquitoes were immobilized by chilling and each mosquito was placed into an individual 30 ml glass vial with screened top and single strip of filter paper on which to rest. Raisins were placed on top of each vial as a sucrose source. After mosquitoes fully recovered and could fly, 2 ml of aged tap water was introduced into the vial for mosquitoes to lay their eggs. Vials were maintained at 26 °C for 5 days, after which vials were filled with ethanol to kill the parent mosquito and the eggs and hatchlings. Eggs and hatchling larvae were counted the same day. Midguts and ovaries from the parent mosquitoes were dissected to quantify blood digestion and ovarian development, based on the Sella scale [25]. For simplicity, Sella stages were combined into two groups: early stages (Sella stages II, III, IV) indicating that minimal vitellogenesis had occurred, and late stages (Sella stages V, VI, VII) indicating that complete or nearly complete vitellogenesis had occurred.

### Feeding on treated mice

Ivermectin stock solution (2000 µg/ml DMSO) was diluted in saline to a working concentration of 80 µg/ml. Three outbred, white mice were weighed and subcutaneously injected with an appropriate volume of ivermectin solution to achieve a dose of 600 µg/kg BW. Three control mice were injected subcutaneously with equivalent volumes of saline. The next day (= 24 h after treatment), each mouse was anesthetized (pentobarbital 60 µg/gm BW, ip) and individually placed onto a screened-top container containing 20–30 *An. albimanus* mosquitoes. Mosquitoes were allowed to feed for ca. 20 min, after which anesthetized mice were immediately transferred to screen-top containers containing 20–30 *An. stephensi* mosquitoes. Mosquitoes were allowed ca. 30 min to feed. Afterwards, mice were returned to the vivarium. Unfed mosquitoes were removed from the cages. Engorged mosquitoes were provided with moistened cotton and a sucrose source (chopped prune), and maintained at 26 °C. Four days after blood feeding, surviving mosquitoes from each mouse were subdivided into aliquots of 3–6 mosquitoes each and gently introduced into new containers containing a cup of water for oviposition. Three days after mice had received injections, a fresh batch of *An. albimanus* and *An*. *stephensi* mosquitoes were fed on the mice and the process described above was repeated. Dead mosquitoes were removed and counted daily for 6 days, after which the number of mosquitoes left alive and total number of eggs laid were counted.

### Data analysis

Mosquito mortalities observed within experimental groups were adjusted for any mortality that occurred within corresponding control groups using Abbott’s formula [26]. Only experimental trials having control mortalities less than 20% were used for further data analyses. Log-probit analyses were conducted on the corrected percent moralities to estimate LC_50_ values (Minitab Inc., State College PA, USA). Mosquito survivorship was analysed with a Kaplan–Meier survival analysis and Log-rank Mantel-Cox test (GraphPad Software, La Jolla, CA, USA). Mosquito fecundities (i.e., average number of eggs laid per female) were compared among treatments using one-way analysis of variance (ANOVA) on log_10_ transformed egg counts. If an ANOVA showed a significant effect due to treatment, the Tukey Honest Significant Differences (Tukey HSD) post hoc test was used to separate statistical differences between groups. Rates of mosquito oviposition and egg hatch, and blood digestion (based on early Sella scores) in treatment groups were compared with their corresponding controls with Chi square analyses (Statistix, Tallahassee FL, USA). A 0.05 level of significance was used throughout.

## Results

### Membrane feeding and intrathoracic inoculations

**Fig. 1:**
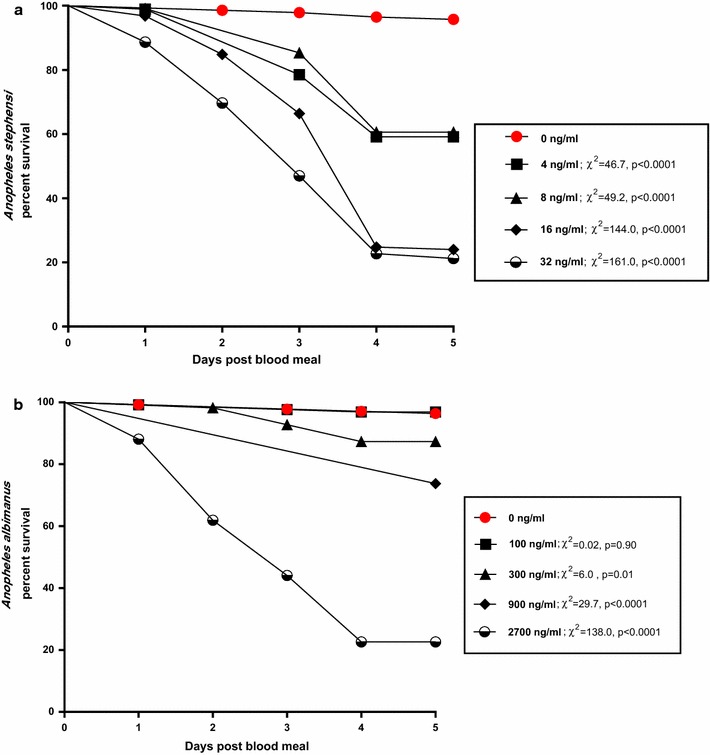
Daily proportion of surviving mosquitoes after ingesting ivermectin at various concentrations. **a** *Anopheles stephensi*, **b** *Anopheles albimanus*. Survival curves for ivermectin-exposed mosquitoes were compared statistically to the survival curves of control mosquitoes using Log-rank Mantel-Cox tests. Test values and p values at each ivermectin concentration are shown

**Fig. 2:**
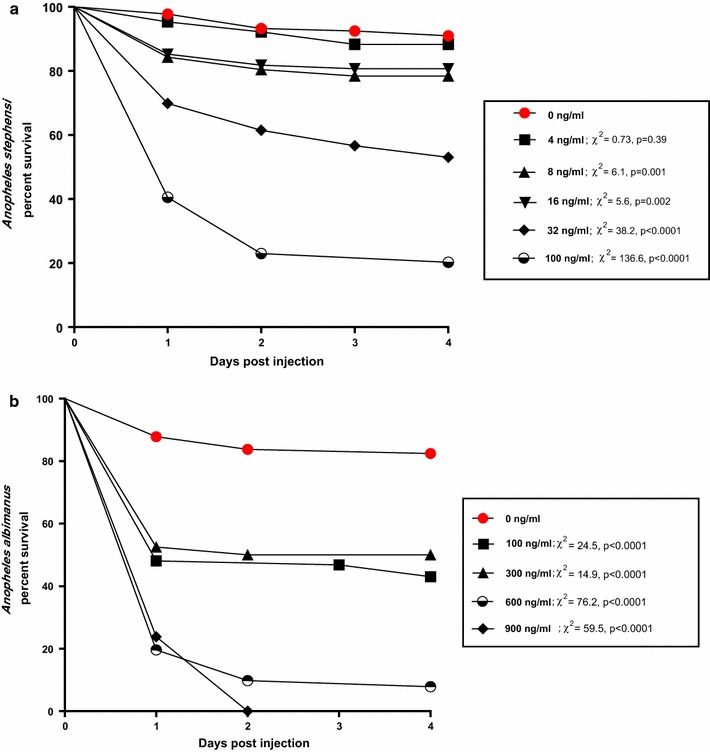
Daily proportion of surviving mosquitoes after intrathoracic inoculation of ivermectin at various concentrations. **a** *Anopheles stephensi*, **b** *Anopheles albimanus*. Survival curves for ivermectin-exposed mosquitoes were compared statistically to the survival curves of control mosquitoes using Log-rank Mantel-Cox tests. Test values and p values at each ivermectin concentration are shown

A total of 573 *An. stephensi* and 582 *An. albimanus* were used to determine the relative oral toxicities of ingested ivermectin. A total of 606 *An. stephensi* and 293 *An. albimanus* were used to determine the relative parenteral toxicities of inoculated ivermectin. When ingested, ivermectin was much more toxic to *An. stephensi* (oral 4-day LC_50_ = 7 ng/ml) than to co-fed *An. albimanus* (oral 4-day LC_50_ = 1468 ng/ml; Table 1). Similarly, intrathoracially-inoculated ivermectin was more toxic to *An. stephensi* (parenteral 3-day LC_50_ = 49 ng/ml) than to *An. albimanus* (parenteral 3-day LC_50_ = 188 ng/ml; Table 1). Lethality of ingested ivermectin did not occur immediately. Instead, mosquito mortality progressed over a period of several days after ingestion of treated blood and treated mosquitoes of both species experienced significant reduction in survivorship over a 4-day period (Fig. 1). Significant reductions of mosquito survival occurred with *An. stephensi* at all doses tested (Fig. 1a) whereas with *An. albimanus,* significant reductions occurred only at the highest doses tested (Fig. 1b). Mosquito mortality occurred more rapidly when ivermectin was inoculated versus when the drug was ingested (Fig. 2).

**Table 1:** Comparative toxicities of ivermectin to *Anopheles* mosquitoes when ingested (oral LC_50_) or injected (parenteral LC_50_)

Species	Oral LC_50_ (95% CL)	Parenteral 3-day LC_50_ (95% CL)	Citation
*An. arabiensis*	7.9 (6.2, 9.9) at 9 days post-feed	–	[14]
*An. stephensi*	*7.0* (*5.2*, *8.6*) *at 4* *days post-feed*	*48.8* (*42.6*, *56.9*)	*present study*
*An. minimus*	16.3 (11.6, 19.4) at 7 days post-feed	–	[15]
*An. gambiae* s.l.	19.8 (± 2.8) at 9 days post-feed	–	[11]
*An. gambiae* s.s.	22.4 (18.0, 26.9) at 5 days post-feed	–	[12]
*An. campestris*	26.4 (21.9, 30.5) at 7 days post-feed	–	[15]
*An. sawadwongporni*	26.9 (24.8, 28.8) at 7 days post-feed	–	[15]
*An. dirus*	55.6 (52.3, 59.1) at 7 days post-feed	–	[15]
*An. aquasalis*	47.0 (44.7, 49.4) at 5 days post-feed	–	[16]
*An. albimanus*	*1468.0* (*1153.5*, *1965.5*) *at 4* *days post-feed*	*187.9* (*136.2*, *239.0*)	*present study*
*An. darlingi*	43.2 (37.5, 48.6) at 7 days post-feed	**–**	[17]

LC_50_ values are expressed as ng/ml. Italic text indicates data resulting from the present study

### Fecundity

The oviposition rate for control-fed *An. stephensi* after ingesting one blood meal (82%, n = 38) was similar to that of control-fed *An. albimanus* after ingesting two blood meals (84%, n = 62) (Table 2). Significant reductions in oviposition rates occurred in *An. stephensi* (33%, n = 6, cχ^2^ = 4.1, p = 0.04) and *An. albimanus* (61%, n = 51, cχ^2^ = 6.5, p = 0.01) that ingested ivermectin at 32 and 1300 ng/ml respectively (Chi square tests, cχ^2 ^≥ 4.1, p < 0.05) but not for mosquitoes that ingested lower concentrations of ivermectin (Table 2, Chi square tests, p > 0.68).

**Table 2:** Reproductive capacity of *Anopheles* mosquitoes ingesting various concentrations of ivermectin via membrane feeder

Mosquito species	Conc (ng/ml)	Total no. surviving females	Oviposition rate of survivors (%)	Geometric mean no. of eggs laid per ovipositing female (95% CI)	Overall hatch rate (%)	Theoretical number of larvae produced per 1000 surviving females
*An. stephensi*	0	38	82^A^	50.1 (34.9, 71.9)^A^	73.3^A^	30,113
	4	14	71^A^	41.6 (22.0, 78.5)^A^	70.1^A^	20,705
	8	12	83^A^	29.8 (13.1, 46.7)^A^	56.1^B^	13,876
	16	12	83^A^	24.7 (15.8, 56.2)^A^	76.0^A^	15,581
	32	6	33^B^	10.4 (2.5, 43.0)^A^	52.4^A^	1798
*An. albimanus*	0	62	84^A^	50.0 (41.0, 60.0)^A^	47.9^A^	20,118
	300	53	87^A^	49.2 (41.2, 60.6)^A^	46.2^A^	19,776
	1300	51	61^B^	39.2 (30.9, 50.0)^A^	18.4^B^	4400

*Anopheles stephensi* mosquitoes were blood fed one time on either treated or untreated blood. *Anopheles albimanus* mosquitoes were blood fed twice; once on untreated blood and again, 2 days later, on either treated or untreated blood. Engorged mosquitoes were held individually in oviposition vials. Superscripts indicate statistically significant differences for parameters within a species

**Fig. 3:**
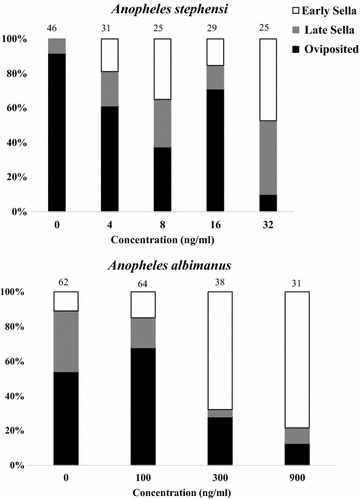
Proportions of *Anopheles stephensi* and *Anopheles albimanus* that completed vitellogensis and successfully oviposited (= Oviposited), underwent extensive vitellogenesis but did not oviposit (= late Sella), or underwent minimal vitellogenesis and minimal blood meal digestion (= early Sella). Mosquitoes were dissected 5 days after ingesting various concentrations of ivermectin via membrane feeding. Numbers above histograms indicate numbers of mosquitoes examined

There were no differences in the average number of eggs laid per ovipositing female between control-fed and ivermectin-fed mosquitoes at any of the dosages tested for both *An. stephensi* (F = 2.05 df = 4, 58; p = 0.10) and *An. albimanus* (F = 1.48, df = 2, 126; p = 0.23) (Table 2). However, the egg hatching rates for *An. stephensi* that ingested 8 ng/ml ivermectin (56%, n = 12, cχ^2^ = 43.1, p < 0.0001) and *An. albimanus* that ingested 1300 ng/ml ivermectin (18%, n = 51, cχ^2^ = 389.1, p < 0.0001) were significantly less than egg hatch rates of the corresponding control-fed groups (73 and 48%, respectively) (Table 2). The egg hatch rate for *An. stephensi* that ingested 32 ng/ml ivermectin (52%) was lower than that of control-fed mosquitoes (73%), but the difference was not quite statistically significant (cχ^2^ = 3.62, p = 0.057). This was likely due to the excessive adult mortality that occurred at this dose and the small number of surviving females (n = 6) left to oviposit (Table 2). By multiplying oviposition rates, fecundity and hatch rates for each treatment group (Table 2, last column), notable reductions in F1 larva production occurred only at doses that exceeded the oral LC_50_ values for each species (see Table 1). Five days after blood feeding, the majority (≥ 90%) of control-fed mosquitoes in both species had either laid their eggs, or were fully gravid in late Sella stages. However with increasing dosages of ivermectin, the proportion of mosquitoes that laid eggs by day 5 diminished (Fig. 3). With exception of *An. albimanus* fed at 100 ng/ml concentration, significantly greater proportions of ivermectin-fed mosquitoes retained undigested blood in their guts and underdeveloped ovaries (early Sella stages), compared to midguts and ovaries observed in control-fed mosquitoes (Fisher’s exact test, p values ≤ 0.007, Fig. 3).

### Feeding on treated mice

One day after mice received injections, there were significant reductions in the 6-day survival rate of *An. stephensi* (15%) and *An. albimanus* (56%) fed on ivermectin-injected mice compared to mosquitoes fed on saline-injected control mice (100 and 89%, respectively, Chi square tests with Yate’s correction, cχ^2^ > 11, p < 0.001; Table 3). Average fecundity in *An. stephensi* fed on ivermectin-treated mice (0.9 ± 1.6 eggs/female) was significantly less than mosquitoes fed on control mice (46.1 ± 25.9 eggs/female) (t test, T = 8.2, df = 13, p < 0.0001). No significant reduction in fecundity was observed for *An. albimanus* fed on ivermectin-treated mice. Three days after mice were treated with ivermectin, there remained a significant, albeit smaller, reduction in the 6-day survival of *An. stephensi* fed on ivermectin-treated mice (62%) *versus* that of *An. stephensi* fed on control mice (96%) (Chi square tests with Yate’s correction, value = 16, p < 0.001; Table 3). No significant difference was observed in the 6-day survival of *An. albimanus* fed on mice 3 days after treatment. By 3 days after mouse injections, there were no significant differences in mosquito fecundity for either species fed on ivermectin-treated versus control mice (Table 3).

**Table 3:** Survival and fecundity of *Anopheles* mosquitoes at 6 days after feeding on ivermectin treated versus untreated mice

Species	Treatment	1 day after mouse injections				3 days after mouse injections			
		Percent surviving (n)		Eggs per female (± SD)		Percent surviving (n)		Eggs per female (± SD)	
*An. stephensi*	Ivermectin	15% (52)	cχ^2^ = 76.5 p < 0.001	0.9 ± 1.6	T = 8.2 df = 13 p < 0.001	62% (66)	cχ^2^ = 15.7 p < 0.001	30.8 ± 8.7	T = 1.0 df = 4 p = 0.373
	Control	100% (56)		46.1 ± 25.9		96% (48)		44.7 ± 18.8	
*An. albimanus*	Ivermectin	56% (56)	cχ^2^ = 11.3 p < 0.001	4.7 ± 8.1	T = 1.5 df = 17 p = 0.143	95% (57)	cχ^2^ = 0.9 p = 0.332	25.2 ± 7.2	T = 0.2 df = 4 p = 0.864
	Control	89% (52)		12.8 ± 13.2		85% (48)		24.8 ± 10.0	

Mice received subcutaneous injections of either ivermectin (600 mg/kg BW) or saline (control group). Three mice were used for each treatment. Cohorts of age-matched mosquitoes (n = sample size) were blood-fed 1 day after injections and 3 days after injections

## Discussion

Ivermectin ingested in a blood meal has been shown to be extremely potent against at least eight different anopheline species that have been tested to-date [11–17]. This study found ingested ivermectin to be similarly potent against *An. stephensi* (Table 1). However, *An. albimanus* is the most ivermectin-tolerant *Anopheles* species evaluated to-date, with an oral LC_50_ over 25-fold greater than other species tested. The relative insensitivity of *An. albimanus* to ingested ivermectin was evident regardless of whether ivermectin was administered in cow blood via membrane feeders or via mosquito feedings on treated mice (Table 3).

To investigate the mechanism(s) of this insensitivity, serial dilutions of ivermectin were injected directly into the haemocoels of *An. albimanus* and *An. stephensi* and the resulting parenteral LC_50_ values were compared with the oral LC_50_ values derived from membrane feeding trials (Table 1). The rationale for injecting ivermectin is that the target molecules for ivermectin (i.e., glutamate-gated chloride channels) reside within nervous and muscle tissue outside of the midgut [27]. Injection bypasses the midgut. When injected directly into the haemocoel of *An. albimanus*, ivermectin not only worked more rapidly (Fig. 2B) but was also tenfold more toxic than when it was ingested in a blood meal (Table 1). This indicates that ingested ivermectin was poorly absorbed across the gut of *An. albimanus*. Even so, injected ivermectin was still significantly less toxic to *An. albimanus* (LC_50_ = 188 ng/ml) than to *An. stephensi* (LC_50_ = 49 ng/ml), suggesting that the binding affinity of ivermectin and/or its stimulatory effect on the glutamate-gated chloride channels of *An. albimanus* was less than that of the glutamate-gated chloride channels of *An. stephensi*. The relative insensitivity of *An. albimanus* to ingested ivermectin may be attributable to (1) poor absorption of ivermectin across the gut of *An. albimanus*, (2) potential structural differences between the glutamate-gated chloride channels among anopheline species, or (3) more efficient detoxification processes occurring in *An. albimanus* compared to other species of *Anopheles* tested.

Curiously, ivermectin was significantly more toxic to *An. stephensi* when ingested (LC_50_ = 7 ng/ml) than when injected (LC_50 _= 49 ng/ml). This suggests that ivermectin metabolites resulting from mosquito digestion of the blood meal may have a contributory role in the overall oral toxicity of ivermectin to *An. stephensi*.

Ingestion of ivermectin also reduced the reproductive potential of *An. albimanus* and *An. stephensi* by inhibiting blood meal digestion, vitellogenesis, oviposition rate, fecundity, and egg hatch (Fig. 3, Tables 2 and 3). However, statistically significant reductions in these reproductive parameters occurred only when mosquitoes ingested ivermectin at or above the respective LC_50_ concentrations for each species. This is similar to that reported for ivermectin in *An. arabiensis* [14] but stands in contrast to results reported for *An. aquasalis* [16], where significant reductions in egg production and hatching were observed after mosquitoes had ingested ivermectin at a substantially lower potency, i.e., a concentration equivalent to the LC_5_ for that species (= 18 ng/ml). Defining the effects of ingested ivermectin on mosquito reproduction is important because significant net reductions in mosquito reproduction could still act to reduce mosquito populations, even at sub-lethal concentrations (see Table 2).

According to the recommendations of most commercial manufacturers of endectocide livestock products, a subcutaneous dose of ivermectin at 200 mg/kg BW is standard for control of intestinal nematodes. At that dose and mode of delivery, the peak plasma concentrations of ivermectin in cattle ranges between 30 and 46 ng/ml [28–30]. Field studies have shown that this amount of ivermectin (200 mg/kg BW) in the blood of treated cattle is sufficient to cause significant mortality and reproductive losses in several species of *Anopheles* mosquitoes, including *An. arabiensis* [31], *Anopheles culicifacies* and *An. stephensi* [32]. This study suggests that plasma levels of ivermectin in this range would not produce any mortality or loss of reproduction in *An. albimanus* that feed on ivermectin-treated cattle.

To successfully implement endectocides for vector control, it is essential to fill the gaps in knowledge about how different mosquito species react to ivermectin. This study found wide differences in oral and parenteral toxicities of ivermectin to the Asian vector, *An. stephensi*, and the Latin American vector *An. albimanus*. These studies utilized long established laboratory strains of mosquitoes. An important next step will be to define comparative toxicities of ivermectin and other endectocides against field strains of *Anopheles* mosquitoes.

## Conclusions

Ivermectin has been shown to be a promising candidate for *Anopheles* vector control in many parts of the world. However, there are several important species of *Anopheles* that have yet to be tested for their susceptibility to ivermectin. This study found that *An. stephensi* was highly susceptible to ivermectin. Conversely, *An. albimanus* was nearly impervious to the effects of ivermectin when compared to other *Anopheles* species tested to date. Significant mortality and sterility of *An. albimanus* were achieved only at ivermectin concentrations so high that they would not be feasible or desirable to use in livestock. Fortunately, there are a number of other related avermectin-based endectocides currently registered for use in livestock in many countries. Further efficacy testing of alternative endectocides should be conducted against Central and South American malaria vectors if endectocides are to be successfully implemented in the Americas for zoophagic vector control.

## Abbreviations

ANOVA: analysis of variance
BW: body weight
DMSO: dimethyl sulfoxide
IP: intraperitoneal
IRS: indoor residual spraying
ITN: insecticide-treated bednet
PBS: phosphate-buffered saline
